# Insights From Long-term Follow-up of a Girl With Adrenal Insufficiency and Sphingosine-1-Phosphate Lyase Deficiency

**DOI:** 10.1210/jendso/bvac020

**Published:** 2022-02-11

**Authors:** Avinaash Maharaj, Tülay Güran, Federica Buonocore, John C Achermann, Louise Metherell, Rathi Prasad, Semra Çetinkaya

**Affiliations:** 1 Centre for Endocrinology, William Harvey Research Institute, John Vane Science Centre, Queen Mary, University of London, Charterhouse Square, London, United Kingdom; 2 Marmara University, School of Medicine, Department of Paediatric Endocrinology and Diabetes, Istanbul, Turkey; 3 Genetics and Genomic Medicine Research and Teaching Department, UCL Great Ormond Street Institute of Child Health, University College London, London, UK; 4 Health Sciences University, Dr. Sami Ulus Obstetrics and Gynecology, Children’s Health and Disease Education and Research Hospital, Ankara, Turkey

**Keywords:** SGPL1, sphingolipids, adrenal insufficiency, steroidogenesis, ovarian calcification

## Abstract

**Introduction:**

Sphingosine-1-phosphate lyase (SGPL1) insufficiency syndrome (SPLIS) is a multisystemic disorder which, in the main, incorporates steroid-resistant nephrotic syndrome and primary adrenal insufficiency (PAI).

**Case Presentation:**

We present a young girl with a novel homozygous variant in *SGPL1*, p.D350G, with PAI in the absence of nephrotic syndrome. In the course of 15 years of follow-up she has further developed primary hypothyroidism and while she has progressed through puberty appropriately, ovarian calcifications were noted on imaging. The p.D350G variant results in reduced protein expression of SGPL1. We demonstrate that CRISPR engineered knockout of *SGPL1* in human adrenocortical (H295R) cells abrogates cortisol production. Furthermore, while wild-type SGPL1 is able to rescue cortisol production in this in vitro model of adrenal disease, this is not observed with the p.D350G mutant.

**Conclusion:**

SGPL1 deficiency should be considered in the differential diagnosis of PAI with close attention paid to evolving disease on follow-up.

Sphingosine-1-phosphate lyase (SGPL1) insufficiency syndrome (SPLIS, nephrotic syndrome, type 14; NPHS14; MIM 617575) is unique among disorders of sphingolipid metabolism due to its multi-endocrine pathology. In addition to steroid-resistant nephrotic syndrome, ichthyosis, and neurological disease, which are seen in some of the other sphingolipidoses, SPLIS also incorporates primary adrenal insufficiency (PAI) and primary hypothyroidism [1]. Furthermore, primary hypogonadism has been reported in affected boys [2-4]. Delineating a clear genotype–phenotype correlation in this disorder is complicated by marked phenotypic heterogeneity since mutation type, domain topology, and perceived enzyme activity do not always predict disease severity.

We report a novel variant in *SGPL1* in a patient presenting with early-onset primary adrenal failure and subsequent hypothyroidism in the absence of renal pathology. Early identification of this syndrome enables ongoing surveillance for the emergence of other disease features allowing timely and appropriate interventions.

## Case Presentation

**Fig. 1.**
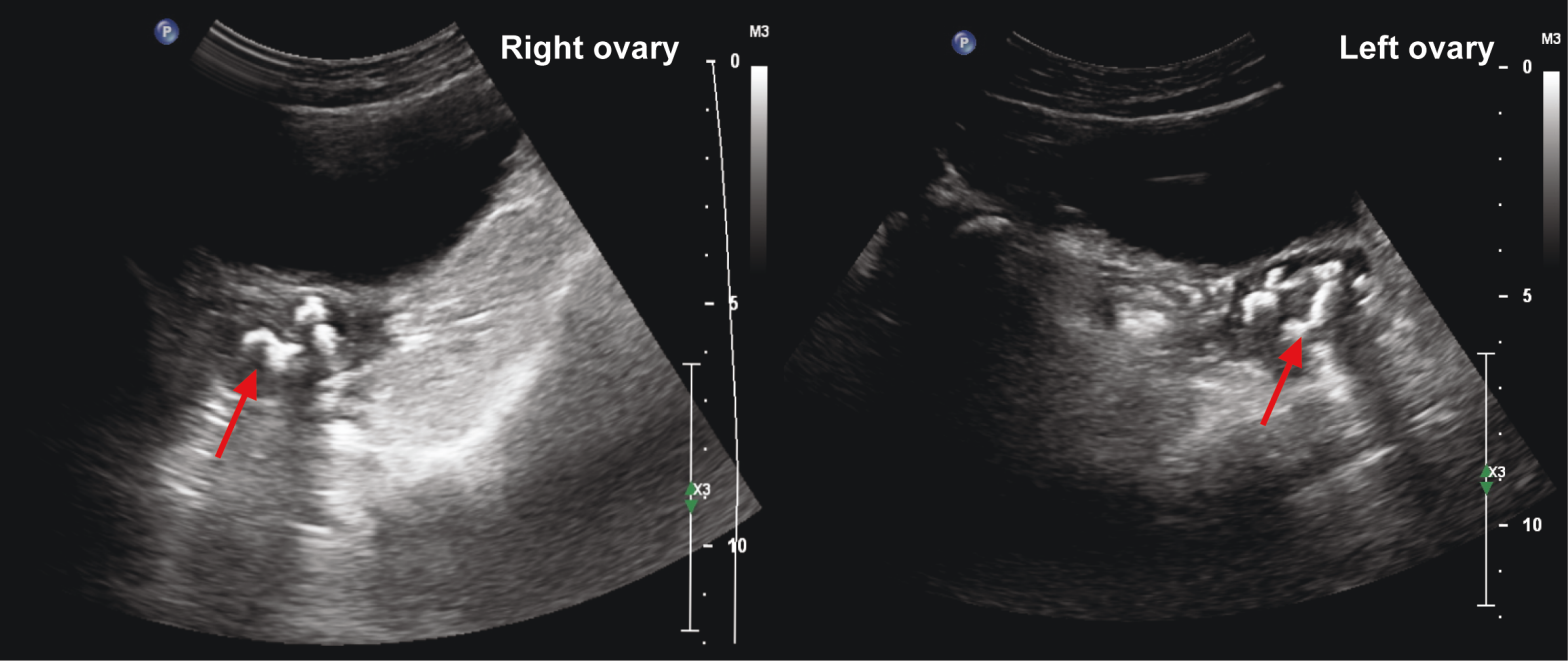
Pelvic ultrasound scan showing areas of calcification (red arrows) in both ovaries.

A Turkish female infant from a consanguineous kindred initially presented at the age of 9 months with fever, seizures, generalized hyperpigmentation, and ichthyosis. Biochemical investigations revealed marked hypocortisolemia (20.9 nmol/L) associated with high plasma ACTH (adrenocorticotropin) (4500 pmol/L) levels. Magnetic resonance imaging revealed increased bilateral parieto-occipital uptake suggestive of meningoencephalitis. Mineralocorticoid deficiency as evidenced by raised plasma renin concentration (55 pg/mL) was also identified and hydrocortisone and fludrocortisone replacement were started. The patient’s recovery was complicated by the development of cortical visual impairment. Since diagnosis, the patient was noted to have steadily increasing thyrotropin levels warranting a diagnosis of primary hypothyroidism treated with L-thyroxine (shown in Table 1). At the age of 10 years, during routine follow-up, a palpable anterior neck swelling was noted. Ultrasonography revealed a nodular 13 × 13 × 5 mm infrahyoid lesion, while thyroid lobe architecture was homogeneous and otherwise unremarkable. Postoperative pathological evaluation was suggestive of a benign thyroglossal cyst with psammomatous calcification. The patient has since entered puberty with normal ovarian reserve (antimüllerian hormone concentration of 4.93 ng/mL, normal range 0.86-10.45) despite detection of ovarian calcifications on imaging conducted for irregular periods(shown in Fig. 1). Renal function, including assessment of urine protein creatinine ratio, during surveillance following diagnosis has been normal (shown in Table 1). Whilst she was born small for gestational age at term (birthweight –2.35 SDS), she had further evidence of growth failure with a final height at –3.67 SDS and parent adjusted height of –2.40 SDS, despite insulin-like growth factor 1 levels within normal range.

**Table 1.** The timeline of pertinent clinical features as they occurred

Age of presentation	Clinical presentation, diagnoses	Biochemistry	Treatment	Radiological findings
9 months	Upper respiratory tract infection, fever, seizures, hyperpigmentation, ichthyosis Diagnosis—meningoencephalitis, primary adrenal insufficiency	WBC: 17.3 × 10^9^/L CRP: 182 mg/L Glucose: 93 mg/dL Na: 136 mmol/L (NR 133-146) K: 4.9 mmol/L (NR 3.5-5.5) BUN: 8 mg/dl (NR 0-23) Creatinine: 0.37 mg/dL (NR 0.6-1.2) ACTH: > 4500 pmol/L (NR 0-103) Cortisol: 0.76 µg/dL (NR 7.2-25) Renin: 54 pg/mL (NR 0.4-33) Aldosterone: 95 pg/mL (NR 70-540) 17 OHP: 0.1 nmol/L (NR < 0.1) DHEASO_4_: <15 µg/dL T. Testosterone: <10 ng/dL Progesterone: <0.1 ng/mL FSH: 4.3 mIU/mL (NR 0-2.4) LH: <0.2 mIU/mL (NR 0-3.8) E2: <5 pg/mL VLCFA levels: Normal	Glucocorticoid and mineralocorticoid replacement, antiviral therapy, anti-epileptic therapy	Cranial MRI-increased bilateral uptake in parieto-occipital leptomeningeal areas
10 years 2 months	Anterior neck swelling		Resection of lesion revealed calcified mass suggestive of thyroglossal cyst	
11 years 5 months	Primary hypothyroidism	TSH: 8.2 mIU/mL (NR 0.6-5.5) Free T4: 10.5 pmol/L (NR 12-24) Thyroid autoantibodies—negative	25 µg/day Na l-thyroxine treatment	Thyroid USS unremarkable
11 years 8 months	Menarche			
13 years				Abnormal pelvic ultrasound scan: Right ovary: 1 calcification area Left ovary: 4 calcification areas (maximum calcification area diameter: 7 mm)
16 year 2 months	Routine follow-up	AMH: 4.93 ng/mL (NR 0.86-10.45) FSH: 6.04 mIU/mL (NR 1.48-11.7) LH: 23.28 mIU/mL (NR 0.6-21) E2: 112.58 pg/mL (NR 13-71) BUN: 10 mg/dL (NR 0-23) Creatinine: 0.67 mg/dL (NR 0.6-1.2) TSH: 1.848 mIU/mL (NR 0.6-5.5) Triglycerides: 86 mg/dL (NR 35-135)		Thyroid USS normal. Ovarian calcifications persist on pelvis USS

Abbreviations: ACTH, adrenocorticotropin hormone; AMH, antimüllerian hormone; BUN, blood urea nitrogen; CRP, C-reactive protein; DHEASO4, dehydroepiandrosterone sulfate; E2, estradiol; FSH, follicle-stimulating hormone; LH, luteinizing hormone; NR, normal range; USS, ultrasound scan; VLCFA, very long chain fatty acids; WBC, white blood cell.

## Materials and Methods

### Variant Detection and Confirmation

A homozygous *SGPL1* variant c.1049A>G;p.D350G was found on whole exome sequencing and confirmed by Sanger sequencing using primers amplifying exon 11 of *SGPL1* (forward: 5′-CATCTTTCCACCCATGTCT-3′ and reverse: 5′- GTGACGGCAAAGAGAGAGT-3′). Pathogenicity of the missense variant was evaluated using a combination of predictive tools: Sorting Intolerant from Tolerant [5], Polymorphism Phenotyping v2 [6], Combined Annotation Dependent Depletion [7], Mutation taster [8], and Protein Variation Effect Analyser [9].

### Protein Structure Modelling and Thermostability Analysis

Protein 3D modelling of Protein Data Bank SGPL1 crystal structure (4Q6R) [10] was performed using the tool PyMOL (Schrodinger, LLC. 2010. The PyMOL Molecular Graphics System, Version X.X) with thermostability of mutant protein assessed using computational platforms: DynaMut [11], I-Mutant2.0-SEQ [12], iSTABLE2.0 (MUpro_SVM, MUpro_NN) [13], iPTREE-STAB [14], and SDM [15].

### Site-directed Mutagenesis

Site-directed mutagenesis of an *SGPL1* (NM_003901) Human Tagged ORF Clone (ORIGENE, RC208705) was performed using the QuikChange II XL site-directed mutagenesis kit (Agilent, 200521) according to the manufacturer’s instructions. Primers for generation of 4 specific mutants in SGPL1 (p.D350G, p.N171D, p.Y15C, and p.F545del) were designed using the online tool https://www.agilent.com/store/primerDesignProgram.jsp.

### CRISPR-Cas9 Engineered Knockout of *SGPL1* in an Adrenocortical Cell Line

CRISPR gene editing was achieved utilizing the protocol outlined by Ran et al [16] and previously published single guide RNA sequences [17]. The single guide RNA oligos were then cloned into pSpCas9(BB)-2A-GFP (PX458), a gift from Feng Zhang (Addgene plasmid #48138; http://n2t.net/addgene:48138; RRID:Addgene_48138, https://www.addgene.org/48138) [16] and introduced into NCI-H295R (H295R) (ATCC® CRL-2128™) adrenocortical cells via transfection using Lipofectamine™ 3000 according to manufacturer’s instructions. After 72 hours, GFP-positive cells were cell sorted by fluorescence-activated cell sorting into prepared 96-well plates, to ensure single cell clonal expansion. Colonies were expanded and genotyped after 4 to 6 weeks.

### Western Blotting

*SGPL1* knockout (KO) H295R cells were seeded into 6-well culture plates and transfected with either wild-type (WT) SGPL1 or mutant construct using Lipofectamine 3000® reagent (Thermo Fisher Scientific). After 48 hours, whole cell lysates were prepared by addition of RIPA buffer (Sigma Aldrich) supplemented with protease and phosphatase inhibitor tablets (Roche). Protein concentrations were quantified using a Bradford protein assay (Bio-Rad) and lysates denatured by addition of Laemmli sample buffer 2× (Sigma Aldrich) and boiled for 5 minutes at 98°C. A 20-µg bolus of protein was loaded into the wells of a 4% to 20% sodium dodecyl sulfate-polyacrylamide gel electrophoresis gel (Novex) prior to electrophoretic separation using MOPS buffer. Protein transfer to nitrocellulose membrane was achieved by electroblotting at 15 V for 45 minutes. The membrane was blocked with 5% fat-free milk in tris-buffered saline/0.1% Tween-20 (TBST) and left to gently agitate for 1 hour. Primary antibody (Human SGPL1 Antibody; AF5535, R&D Systems; RRID:AB_2188674, http://antibodyregistry.org/AB_2188674) was added at a concentration of 1:1000 with mouse anti-Actin beta monoclonal antibody (Abcam, ab6276, RRID:AB_2223210, http://antibodyregistry.org/AB_2223210) at a concentration of 1:10 000 used as a housekeeping control. Primary antibody incubation was left overnight at 4°C with gentle agitation. The membrane was then washed for 5 minutes (3 times) with TBST. Secondary antimouse (IRDye® 800CW Goat antimouse IgG; RRID:AB_10793856, http://antibodyregistry.org/AB_10793856) and antigoat (IRDye® 680RD donkey antigoat IgG; RRID:AB_10956736, http://antibodyregistry.org/AB_10956736) antibodies were added at a concentration of 1:5000 to blocking buffer and the membrane incubated at 37°C for 60 to 90 minutes. The membrane was subsequently washed 3 times (5 minutes each) with TBST and visualized with the LI-COR Image Studio software for immune-fluorescent detection.

### Forskolin Stimulation of Adrenocortical Cells

*SGPL1* WT and KO H295R cells were seeded into 6-well plates at a density of 2.5 × 10^6^ cells per well and cultured in serum-free media. Additionally, discrete wells were transfected (using Lipofectamine™ 3000 according to the manufacturer’s instructions) with *SGPL1* ORF clone (WT) and p.D350G mutant constructs. Cells were then stimulated with 10 µM forskolin for 24 hours followed by harvesting of media and protein extraction from cells. Forskolin treatment, as a surrogate to ACTH stimulation, has been shown to alter expression of steroidogenic enzymes in NCI-H295R cells leading to an almost 25-fold increase in cortisol production [18]. Cortisol levels in supernatants were analyzed on a Roche Modular E170 automated immunoassay analyzer using electrochemiluminescent detection as per previous publication [19]. Cortisol measurements were normalized to protein levels following protein quantification by Bradford assay. Data are presented as the mean ± SD of 3 experiments.

### Statistics

Statistical analysis was performed using a 2-tailed Student’s t test to generate *P* values. *P* ≤ .05 was considered statistically significant. Data are presented as mean ± SD where error bars are shown.

## Results

**Fig. 2.**
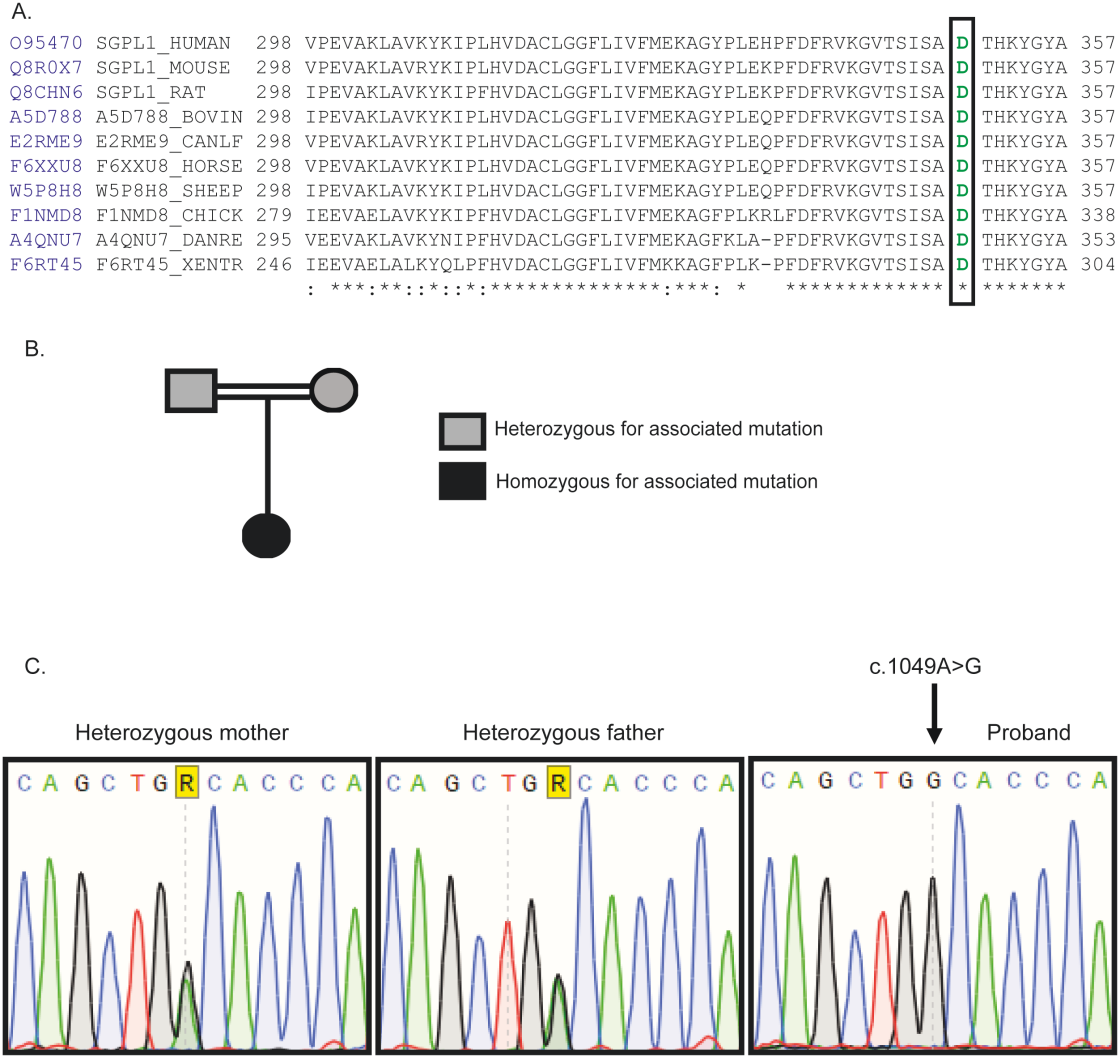
(A) Partial alignment of SGPL1 protein sequences, showing conservation of aspartic acid (D) at position 350 (green) across several species. Sequence conservation is beneath the alignment; asterisks, total conservation:partial conservation. (B) Pedigree of affected patient. Black-filled symbol indicates our patient, homozygous for SGPL1 (c.1049A>G, p.D350G). Gray-filled symbols indicate the parents, heterozygous for the mutation. (C) Partial sequence chromatograms of genomic DNA from asymptomatic heterozygote parents and the homozygote patient showing the base change from A to G in exon 11.

**Fig. 3.**
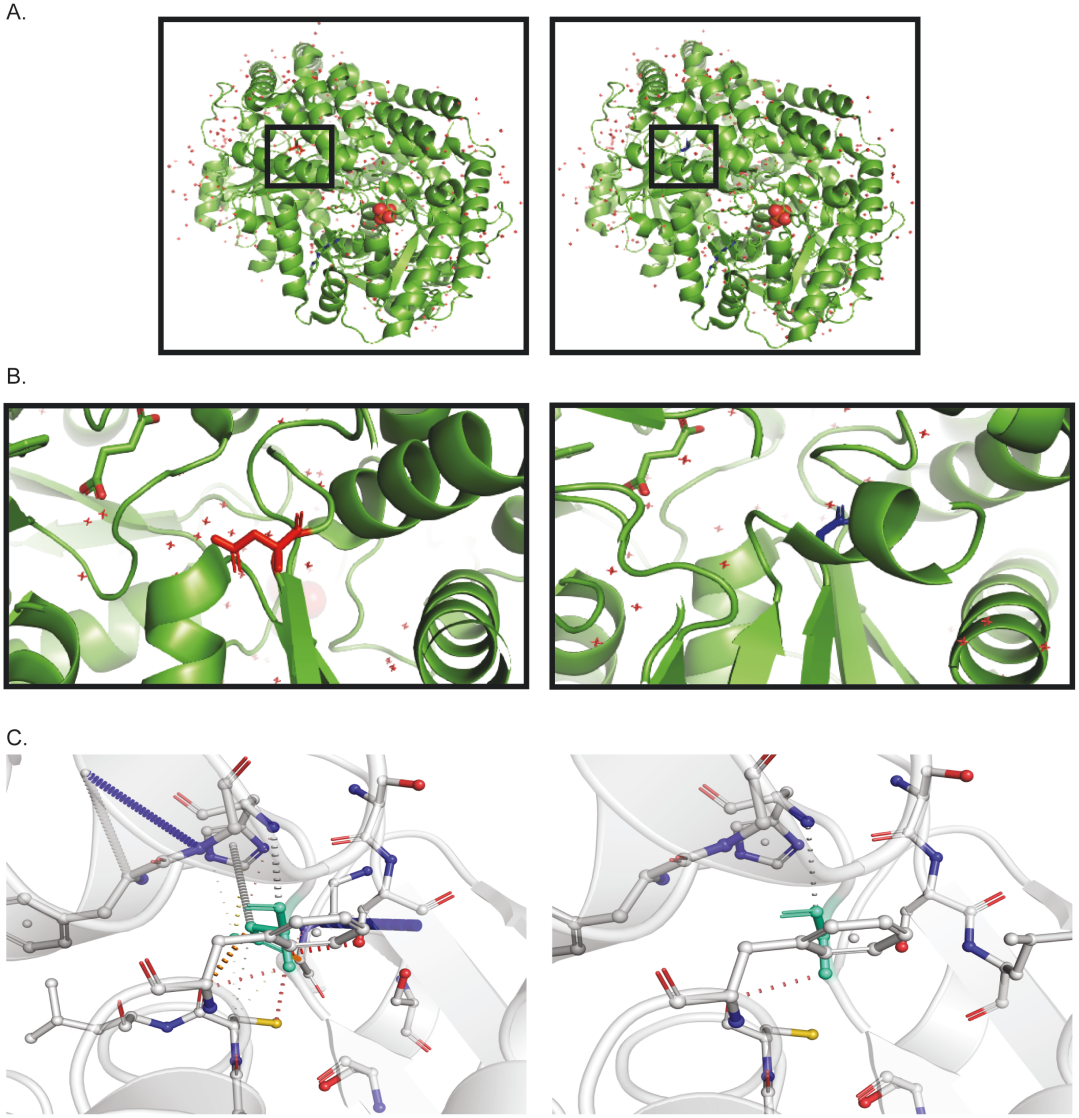
(A) Substitution of the charged aspartic acid for the smaller glycine at codon 350 is demonstrated by modeling, where aspartic acid is presented as red and glycine as blue. (B) Inset showing magnification of the amino acid substitution. (C) This amino acid change disrupts the native molecular bonds rendering the ensuing mutant unstable.

**Fig. 4.**
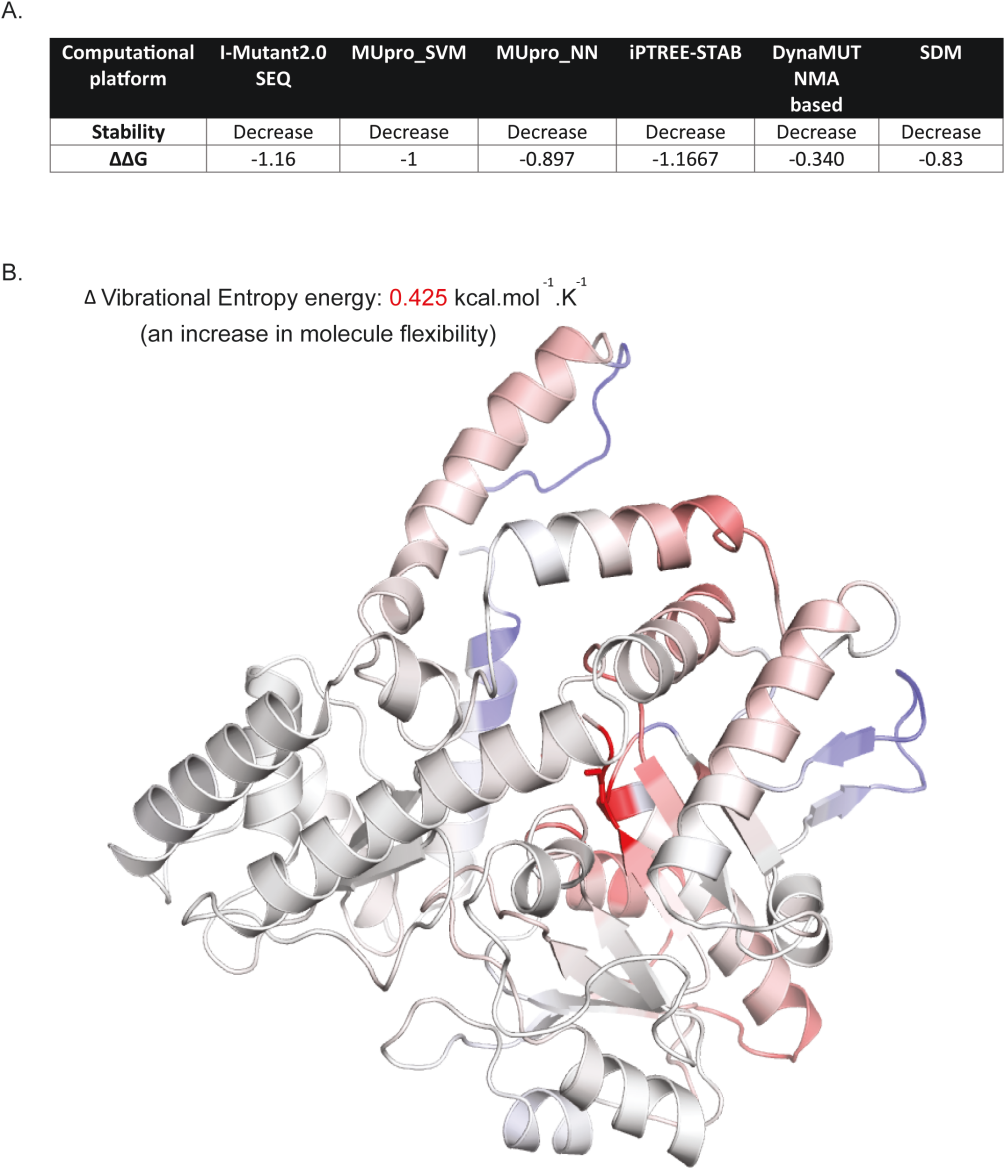
(A) Substitution of glycine for aspartic acid at position 350 renders the protein thermally unstable as demonstrated across several predictive computational platforms. (B) Flexibility analysis using DynaMut suggests p.D350G increases structural mobility.

**Fig. 5.**
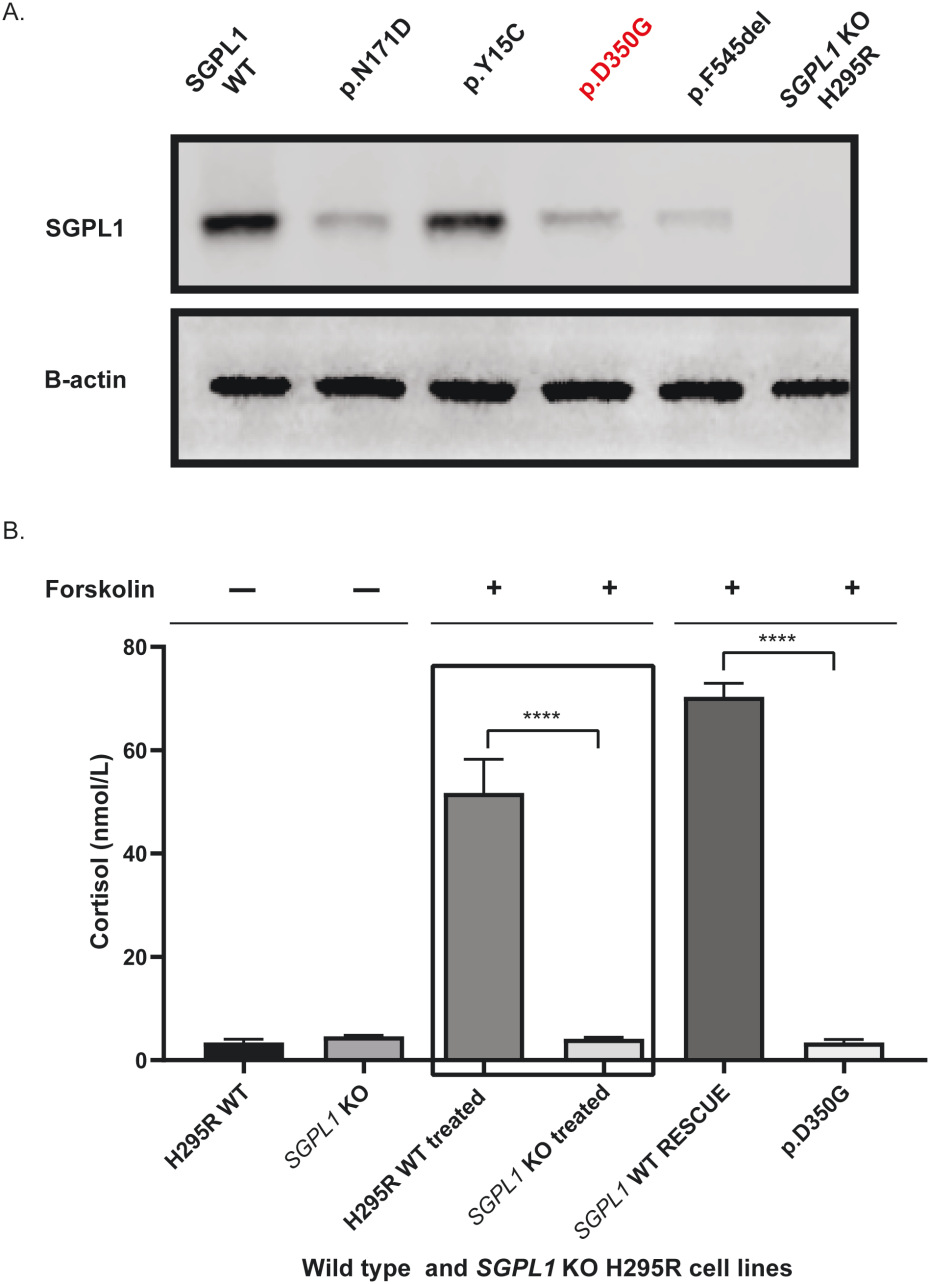
(A) Immunoblotting of SGPL1 in mutant constructs. Representative Western blot showing lower levels of SGPL1 in p.D350G and other mutant constructs except for p.Y15C, which demonstrated a subtle reduction in protein expression. (B) Cortisol output of wild type and SGPL1 KO H295R cell lines after forskolin stimulation. Electrochemiluminescent assay cortisol measurements (normalized to protein content mg/mL) show a robust response in wild-type cells compared with SGPL1-knockout. Markedly less steroid output was obtained from expression of the p.D350G variant compared with wild-type rescue. Data are presented as the mean ± SD of 3 repeated measurements (3 independent replicates) (****P < .0001).

Whole exome sequencing of patient DNA revealed a novel homozygous variant in *SGPL1* (chr10:72631733A>G, c.1049A>G), which was confirmed by Sanger sequencing. Multiple protein sequence alignment across several mammalian species showed evolutionary conservation of the aspartic acid at position 350 of SGPL1 (shown in Fig. 2A). Both parents were heterozygous for this variant (shown in Fig. 2B and 2C). The concordance among predictive platforms in assigning pathogenicity of this variant was high, with p.D350G predicted to be deleterious across the 5 computational platforms utilized. Protein modelling using PyMOL (shown in Fig. 3A and 3B) and DynaMut (shown in Fig. 3C) demonstrated significant alterations to protein conformation, with substitution of glycine for aspartic acid at position 350 predicted to lead to thermal instability (ΔΔG < 0) and increased molecule flexibility (shown in Fig. 4A and 4B). The effect of this variant was evaluated alongside other known published mutations (p.N171D, p.Y15C, and p.F545del) [2, 20, 21]. Patients with *SGPL1* mutations p.N171D and p.F545del presented with both PAI and nephrotic syndrome, while the patient with p.Y15C variant was atypical, presenting with neurological disease alone. SGPL1 protein levels probed by immunoblotting revealed decreased expression of p.D350G and the other mutants when compared with WT; with the highest expression in p.Y15C correlating with the milder/atypical phenotype of this patient (shown in Fig. 5A). To further test the function of the p.D350G variant, CRISPR engineered *SGPL1*-KO human adrenocortical (H295R) cells were utilized as a vehicle for corticosteroid measurement following forskolin stimulation. Basally, both WT and KO cells showed minimal cortisol output but following forskolin treatment, *SGPL1*-KO cells showed no response compared to a brisk response in WT cells (shown in Fig. 5B). Cortisol measurement of cell sera following transfection with *SGPL1*-WT or the p.D350G variant construct revealed an inability of the mutant to rescue cortisol output in contrast to the *SGPL1*-WT construct (shown in Fig. 5B).

## Discussion

Mutations in *SGPL1* often produce highly variable clinical phenotypes, ranging from severe multisystemic disease, associated with fetal hydrops and early mortality, to isolated single organ disease [2-4, 22]. This further complicates establishing a strong genotype–phenotype correlation for SPLIS. Patients with this disorder of sphingolipid metabolism almost invariably present with congenital or steroid-resistant nephrotic syndrome, often progressing to end-stage renal disease [23]. Our patient, with the p.D350G mutation in a highly conserved domain of the lyase, is one of the exceptions, presenting primarily with adrenal failure. She had ichthyosis and thyroid disease, also described in this rare syndrome. Four other individuals with SPLIS in the literature have presented with adrenal insufficiency without concomitant nephrotic disease, all with the p.R222Q *SGPL1* mutation [2, 24]. While 2 of these individuals had other clinical features associated with the syndrome including lymphopenia and seizures [2, 24], 2 presented with adrenal disease alone [2], highlighting the importance of considering SGPL1 in the differential diagnosis of isolated primary adrenal insufficiency.

Impaired steroid production in SPLIS is also evident in the gonad, with approximately one-third of affected males presenting with primary gonadal insufficiency [2-4, 20, 25, 26]. Ovarian pathology has not been previously reported. An impact on puberty has not been widely studied in this condition, particularly in girls, often precluded by the high early-childhood mortality associated with SPLIS. Certainly both sexes of *Sgpl1*^*–/–*^ mouse models are sterile, indicating an effect of SGPL1 deficiency on both ovarian and testicular function [27], likely due to an accumulation of sphingosine-1-phosphate [27, 28]. In *Sgpl1*^*–/–*^mice, postnatal reduction in testis size, loss of spermatocytes in the testis cords, and loss of spermatogenesis are observed together with reduced expression of steroidogenic enzymes. Similarly *Sgpl1*^*–/–*^postnatal ovaries have reduced steroidogenic enzyme expression and are noted to be smaller with fewer antral follicles and corpora lutea [27]. Our patient demonstrated normal pubertal development; this has also been described in 1 other female patient in the literature with typical SPLIS features [2]. However, our patient has developed non-neoplastic ovarian calcifications of unknown significance, which do not at this stage appear to be impeding gonadal function. There is clearly a need for surveillance of patients of both sexes throughout puberty and beyond, given the potential risk of evolving gonadal insufficiency. Interestingly, adrenal calcifications have been reported in several SPLIS patients [3, 4, 20, 22, 25]. While the exact mechanism for this is unknown, adrenal calcifications seen with lysosomal acid lipase deficiency or Wolman disease are due to cholesterol and fatty acid deposition within the adrenal cortex [29], suggesting that calcification in this sphingolipidosis may be due to lipid dyshomeostasis. There are no other reports in the SPLIS literature of calcified thyroglossal cysts, as is reported with our patient and this may be an incidental finding unrelated to the syndrome. Our patient was born small for gestational age with further poor growth on follow-up despite normal insulin-like growth factor 1 levels. SGPL1 deficiency in murine models is associated with significant postnatal growth restriction [27]; however, there are limited data on growth within the SPLIS patient cohort, impeded by high early mortality and lack of follow-up data.

Given the high burden of disease and implications of a positive SPLIS diagnosis, in silico predictive tools may be useful as an initial step to delineate which variants may be disease causing, although functional testing remains the gold standard in evaluating pathogenicity. Thermodynamic profiling designated p.D350G as a destabilizing variant (ΔΔG < 0, shown in Fig. 4A), suggesting that this point mutation renders the protein unstable due in part to loss of hydrogen bonding (Fig. 4B). The mutagenized p.D350G construct exhibited reduced SGPL1 protein expression when compared with WT SGPL1. Other published variants (p.N171D, p.F545del, and p.Y15C) [2, 20, 21] demonstrated similarly reduced expression, with the exception of p.Y15C. The subtle reduction in protein mirrored the mild phenotype of isolated neurological disease seen with the p.Y15C variant and increased protein stability on thermodynamic profiling prediction; indeed, the absence of typical features of SPLIS in this patient were interpreted with the caveat that they may be attributable to another genetic defect [21].

The degree of residual enzymatic activity should theoretically predict phenotypic severity. Prasad et al expressed mutagenized constructs for missense variant p.R222Q and in-frame deletion, p.F545del in *Sgpl1*^–/–^ mouse embryonic fibroblasts and measured hexadecanal production as a surrogate marker of lyase activity [2]. Despite significantly abrogated enzyme activity for both pathogenic variants, the phenotype of each patient was different. p.F545del rendered a more striking phenotype with rapid disease progression while p.R222Q demonstrated marked variability among affected family members harboring the same mutation [2].

Reduced steroidogenic output associated with mitochondrial dysfunction is observed in SPLIS patient–derived dermal fibroblasts with disordered sphingolipid metabolism [30]. We have established *SGPL1*-KO H295R cells as an in vitro adrenal model of the disease. Despite forskolin stimulation for 24 hours, *SGPL1* KO H295R cells demonstrated an impaired cortisol response compared with control. Forskolin stimulation of the KO cells transfected with the mutagenized *SGPL1* D350G construct similarly demonstrated diminished cortisol responsiveness compared with a WT rescue. Interrogation of this in vitro model disease is currently underway to explore the precise impact that SGPL1 deficiency has on steroidogenesis.

In conclusion, our patient presented atypically with adrenal disease in the absence of nephrotic syndrome, despite the p.D350G variant resulting in markedly reduced SGPL1 expression. A clear functional effect was demonstrated in an in vitro adrenal model of disease. Genetic modifiers and other tissue-specific pathogenic mechanisms including secondary effects of sphingolipid accumulation and dysregulated signaling pathways may account for the lack of phenotypic uniformity seen with ablation of SGPL1 activity. The published literature suggests that the prognosis for these patients is poor with just under 50% mortality in childhood, the majority of those deaths occurring in the first year of life [20]. Future research will potentially lead to targeted genetic therapies but, for now, a heightened awareness of the syndrome will allow earlier recognition, enabling appropriate and timely intervention targeted towards limiting morbidity and improving quality of life outcomes.

## Data Availability

Some or all datasets generated during and/or analyzed during the current study are not publicly available but are available from the corresponding author on reasonable request.

## Abbreviations

ACH: adrenocorticotropin
ACTH: adrenocorticotropin hormone
KO: knockout
PAI: primary adrenal insufficiency
SGPL1: sphingosine-1-phosphate lyase
SPLIS: sphingosine-1-phosphate lyase insufficiency syndrome
WT: wild type
